# Dual-Ion Based Magneto-ionic Effects in Nanoporous Pd_75_Co_25_ Alloy

**DOI:** 10.1021/acsmaterialsau.5c00245

**Published:** 2026-03-20

**Authors:** Stefan Eber, Georg Haberfehlner, Peter Banzer, Roland Würschum, Stefan Topolovec

**Affiliations:** † Institute of Materials Physics, 27253 Graz University of Technology, NAWI Graz, Petersgasse 16, 8010 Graz, Austria; ‡ Institute of Electron Microscopy and Nanoanalysis, 365357 Graz University of Technology, NAWI Graz, Steyrergasse 17, 8010 Graz, Austria; ¶ Institute of Physics, 27267 University of Graz, NAWI Graz, Universitätsplatz 5, 8010 Graz, Austria

**Keywords:** magneto-ionics, magnetic materials, magnetic properties, dealloying, nanoporous materials, electrochemistry

## Abstract

Magneto-ionics is an energy-efficient approach to control the magnetic properties of a material by the application of a voltage. For instance, a voltage-induced switching between a ferromagnetic ON state and a magnetic OFF state can be achieved in Co by the reversible reduction/formation of Co (hydr-)­oxides. In this work, we demonstrate that, in addition to this magneto-ionic effect based on oxygen species, a magneto-ionic effect based on hydrogen can be obtained by changing from pure Co to a Pd–Co alloy. To this end, we prepared a nanoporous Pd_75_Co_25_ alloy with a porous structure in the 10 nm range by dealloying of Al_80_(Pd_75_Co_25_)_20_. Initially, the nanoporous alloy is mostly oxidized and thus generates only a very weak magnetic signal, corresponding to an OFF state. Inducing the reduction of the Co (hydr-)­oxides by applying a voltage results in a transition to a ferromagnetic ON state. Due to the macroscopic sample size, the ON state exhibits a considerable absolute magnetic moment in the order of 0.01 emu. This moment can be obtained within just a few minutes thanks to the nanoporous structure. A detailed electrochemical characterization reveals that the magnetization of the alloy is also significantly influenced by the absorption of hydrogen into the nanoporous material, which becomes apparent through an additional increase in the magnetization during the hydrogen desorption. This second magneto-ionic effect is faster and occurs within the time frame of seconds. Overall, the occurrence of these two magneto-ionic effects, which are based on different kinds of ionic species, opens up the possibility to adapt the magneto-ionic response by adjusting the composition of the Pd–Co alloy.

## Graphical abstract

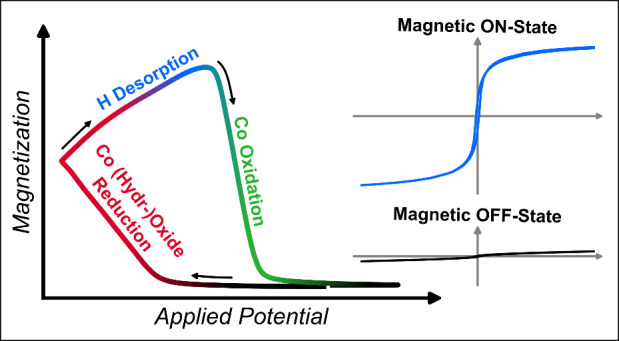

## Introduction

Over the last years, the voltage control of magnetism by electrochemical (surface) reactions and ionic motion has been a rapidly growing research field.^1–5^ This is due to the fact that such magneto-ionic effects combine the advantages of nonvolatility, room-temperature operation, low energy consumption, and the suitability to affect also the bulk magnetism of materials exhibiting a high magnetization.^4,5^ Thus, magneto-ionics is a highly attractive magnetoelectric approach for the usage not only in spintronic devices and magnetic memories, but also for neuromorphic computing, sensor applications, and switchable, energy-efficient (micro)­magnets.^2,3,6,7^

The magnetic material that has been most frequently investigated with respect to magneto-ionic effects is Co, where mainly the reduction of Co (hydr-)­oxides was used to induce changes in the magnetic properties, like the magnetization or coercivity/anisotropy, ideally even a switching between a ferromagnetic and a paramagnetic state.^7–27^ The disadvantage of this magneto-ionic effect, using the oxidation of Co/reduction of Co (hydr-)­oxides, is the limitation of this process to surface near regions of the magnetic material. Thus, these studies have mainly investigated thin films with thicknesses in the nanometer range,^7–18,21–27^ except for two examples focusing on other geometries exhibiting high surface-to-volume ratios, i.e., Co nanopillars^19^ and a porous α-Co­(OH)_2_ film with a thickness of 0.5 mm.^20^

In addition to the aforementioned studies, in which Co or its respective (hydr-)­oxide were in direct contact with a solid or liquid electrolyte, several publications in recent years have dealt with magneto-ionic effects in material systems consisting of a Co phase and a Pd phase. The incorporation of hydrogen into these systems occurred via the electrolyte that was in contact with the Pd phase. For instance, Pd/Co (multi)­layer systems were investigated, where it was demonstrated that changes in the anisotropy, a transition from perpendicular to in-plane anisotropy, and modifications in the coupling between the layers can be induced in this way.^15,28–32^ Another example are works by Goessler et al.,^33,34^ showing that the magnetization of superparamagnetic Co-rich clusters embedded in a Pd matrix can be significantly enhanced by a hydrogen induced change of the RKKY interaction.

Since the Co itself was not in direct contact with the electrolyte in all these Pd–Co systems, only hydrogen based magneto-ionic effects could be observed. However, it seems promising to achieve a magneto-ionic effect based on the reduction/oxidation of Co (hydr-)­oxides also in Pd–Co systems, provided that the Co is in direct contact with the electrolyte, as described for the pure Co systems above. This way, magneto-ionic effects based on both, oxygen and hydrogen ionic species, would be obtainable in the same material. Such dual-ion magneto-ionic effects were, so far, mainly observed in oxides that exhibit ferromagnetism only at low temperatures (see, e.g., refs 35 and 36) and were just very recently reported for Ni films,^37^ i.e., for a room-temperature ferromagnet. To achieve this, rather than utilizing Pd–Co systems, in which the two phases are separated, we aim for a Pd–Co alloy. Due to the combination of oxophilic Co and hydrophilic Pd, both reactions, hydrogen absorption/desorption and reduction/formation of Co surface (hydr-)­oxides, can be induced electrochemically in such an alloy.^38,39^ A corresponding increase/decrease in the magnetization can be anticipated for the latter process, as was observed for the pure Co systems described above. Additionally, variations of the magnetization due to H absorption/desorption can also be expected for Pd–Co alloys, as demonstrated by experiments in which H was incorporated from the gas phase.^40–42^ So far, magneto-ionic effects in Pd–Co alloys induced by oxygen and hydrogen ionic species have not been reported in literature. However, two published articles report on the influence of what was considered as the charging of the electrochemical double layer on the magnetization of Pd–Co alloys, observing a small variation of <5%.^43,44^

To fabricate a macroscopic Pd–Co alloy sample with a high surface area, enabling large absolute magnetic moment variations, we decided to prepare nanoporous (np) Pd–Co alloy samples by dealloying. The utilization of such macroscopic np samples for magneto-ionics offers a distinct advantage over alternative macroscopic samples. In the latter, the times required to induce the magnetism variations are in the range of several hours,^45,46^ whereas significantly lower times can be obtained when using np structures.^33,34,47,48^

In this work, we show that the magnetization of a np Pd_75_Co_25_ alloy can be substantially altered within a timespan of minutes, thereby corresponding to an ON/OFF switching of ferromagnetism. We reveal that this large, reversible variation in magnetization arises primarily from the reduction/formation of Co (hydr-)­oxides. However, it is also demonstrated that the magnetization is additionally influenced by the absorption/desorption of hydrogen into/from the alloy sample, i.e., that magneto-ionic effects based on hydrogen and oxygen species can be induced in Pd–Co alloys, which opens up the possibility to systematically adapt the magneto-ionic behavior by adjusting the oxophilic Co and hydrophilic Pd content of the alloy in the future.

## Experimental Section

### Materials

KOH pellets (Carl Roth, ≥ 85% p.a.) and high-purity water (Carl Roth, ROTIPURAN) were mixed to prepare aqueous electrolyte solutions of different concentrations. The precursor alloys for dealloying Al_80_(Pd_75_Co_25_)_20_, Al_80_Pd_20_, and Al_80_Co_20_ were made out of an Al wire (99.95%, Chempur), a Pd wire (99.95%, Goodfellow), and a Co wire (99.998%, Chempur). The working and counter electrodes were contacted with gold wires (Ø 0.1 mm, 99.9%, Chempur). Ag/Ag_2_O quasi-reference electrodes were fabricated by utilizing a Ag wire (Ø 0.25 mm, 99.995%, Chempur).

### Preparation of Precursor Alloys

The precursor alloys Al_80_(Pd_75_Co_25_)_20_, Al_80_Pd_20_, and Al_80_Co_20_ were melted in an arc melter (MAM-1, Edmund Bühler) under Ar atmosphere, using the Al, Pd, and Co wires in the corresponding fractions. A Ti getter was used to further reduce the presence of oxygen during the melting process. The melt was solidified into rods of 3 mm diameter by suction casting. For dealloying, the rod was cut into 0.5 mm thick discs, which were then halved.

### Dealloying and Electrochemical Activation

The semicircle shaped samples were tightly wrapped with a gold wire to establish an electrical contact. For the production of the np Pd_75_Co_25_ samples, a dealloying procedure based on the work of Xu et al.^49^ was used. The Al_80_(Pd_75_Co_25_)_20_ discs were immersed in a 5 M KOH aqueous solution. After 4 h, no further gas evolution was observed, indicating the completion of the Al dissolution. Afterward, the dealloyed samples were placed in high-purity water for 1 h to remove residuals. In a next step, the samples were activated by performing cyclic voltammetry in an electrochemical cell with a three-electrode configuration (see below). Five consecutive cycles with voltage limits set at – 1.25 and 0.55 V were conducted. The same procedure, but starting with the Al_80_Pd_20_ or Al_80_Co_20_ precursor alloy, was applied to produce the np Pd and np Co samples, which were used for the purpose of comparison.

### Electrochemical Measurements

All electrochemical measurements were carried out in a three electrode setup using a PGSTAT204 potentiostat (Metrohm Autolab). The np samples acted as the working electrode, a high surface area activated carbon cloth was used as counter electrode, and a 1 M KOH aqueous solution was utilized as the electrolyte. Ex situ measurements were performed with a Ag/AgCl reference electrode (Metrohm, 3 M KCl), whereas in situ experiments in the magnetometer were conducted with a Ag/Ag_2_O quasi-reference electrode. It should be noted that there is a potential shift of approximately 100 mV between both reference electrodes, and that the potentials of the measurements were adapted accordingly. The stability of the quasi-reference electrodes in a 1 M KOH aqueous solution was verified with an open circuit potential measurement with respect to a Ag/AgCl reference electrode (see Figure S1 in the Supporting Information). Cyclic voltammetry experiments were all performed with a scan rate of 0.5 m V s^–1^.

### Preparation of Ag/Ag_2_O Quasi-reference Electrodes

Ag/Ag_2_O quasi-reference electrodes were prepared by immersing a Ag wire as working electrode in a 1 M KOH solution, with a second Ag wire as counter electrode and a Ag/AgCl reference electrode (Metrohm, 3 M KCl). The Ag wire working electrode was oxidized by increasing the voltage linearly from the open circuit potential to a maximum of 0.5 V at a scan rate of 5 m V s^–1^.

### Electrochemical Cell Design for Magneto-ionic Studies

For the in situ electrochemical measurements in the SQUID magnetometer, an electrochemical cell setup, developed and tested by our group,^33,34,50^ was applied and further modified. The cell was assembled inside a 17.8 cm long borosilicate glass NMR tube (Ø 5 mm) (see Figure S2 in the Supporting Information). The np Pd_75_Co_25_ working electrode was positioned approximately at the middle of the tube, in close proximity to the activated carbon counter electrode. The Ag/Ag_2_O quasi-reference electrode was placed at the bottom of the NMR tube, which was filled with a 1 M KOH aqueous solution. To prevent short circuits, all electrodes were isolated using polyethylene tubing. In the end, the glass tube was sealed with a polypropylene plug, containing three holes for the electrode wires, and a two-component epoxy adhesive (UHU Plus Endfest). With the exception of the working electrode, no ferromagnetic materials were utilized in order to minimize other contributions to the measured magnetic signal.

### SQUID Magnetometry

Apart from ex situ magnetization curve measurements, all magnetic experiments were performed using a SQUID magnetometer (Quantum Design, MPMS-XL-7). For Zero Field Cooling and Field Cooling (ZFC/FC) measurements, np Pd_75_Co_25_ samples were placed in polycarbonate capsules. The remaining space was filled with cotton to prevent any sample movement during measurements. The capsules were sewn into a sample holder straw and subsequently inserted into the magnetometer, where a constant magnetic field of 0.005 T was applied and the temperature was varied between 300 and 4.2 K. For in situ experiments, the electrochemical cells were mounted on a sample transfer rod, providing electrical connections for all three electrodes with the potentiostat. During all in situ measurements, the temperature was maintained at 300 K, and, with the exception of in situ magnetization curve experiments, a constant magnetic field of 0.5 T was applied. At this field, a saturation of the ferromagnetic magnetization curve of np Pd_75_Co_25_ was observed.

### VSM

Ex situ magnetization curves were recorded with a Vibrating Sample Magnetometer (Lake Shore, VSM Series 8600). For these measurements, np Pd_75_Co_25_ samples were placed in polycarbonate capsules, which were filled with cotton and sewn into sample holder straws.

### SEM, STEM, EDX

Np Pd_75_Co_25_ samples prepared by dealloying were mortared to a fine powder and transferred on a TEM grid via drop casting. Representative particles underwent imaging and were subjected to a quantitative elemental analysis. Scanning Transmission Electron Microscopy (STEM) and Energy Dispersive X-ray Spectroscopy (EDX) were performed with a FEI Titan^3^ G2 60–300 microscope using a Super-X 4-quadrant EDX detector. It was operated at 300 kV with a beam current of 100 pA and a convergence angle of 19.6 mrad. A camera length of 115 mm was used, corresponding to a collection angle from 50 to 200 mrad for High-Angle Annular Dark Field (HAADF) imaging. For the SEM (Scanning Electron Microscopy) investigation, a Zeiss Ultra 55 FEG SEM was used. Surface sensitive images of the np structure were obtained with a low acceleration voltage of 5 keV. The High-Definition Angular selective Backscattered (HDAsB) electron detector was employed for backscattered electron imaging.

### XRD

The XRD analysis was performed with a Rigaku Miniflex 600 utilizing a D/teX Ultra2 high-speed one-dimensional silicon strip detector. Several np Pd_75_Co_25_ samples were finely ground into a powder and positioned on a Si sample holder. The peaks were assigned using the Powder Diffraction File (PDF) database.

## Results and Discussion

For the dealloying procedure, an Al_80_(Pd_75_Co_25_)_20_ precursor alloy was fabricated and samples were immersed in 5 M KOH to receive a np Pd_75_Co_25_ alloy. This composition was chosen, because Pd–Co alloys with such a Co fraction have a Curie temperature that is significantly higher than room temperature,^51^ which should consequently result in a ferromagnetic behavior. Moreover, Pd–Co alloys of such a composition exhibit the capacity for hydrogen insertion.^52^

### Structural and Elemental Analysis

To investigate and visualize the structure obtained by dealloying the Al_80_(Pd_75_Co_25_)_20_ precursor alloy, STEM measurements were performed. The resulting images are shown in Figure 1a and b, which reveal a highly porous nature with pore sizes of around 10 nm. This porous structure accounts for the majority of the investigated sample, only a small part (marked as area #3 in Figure 1a) appears to be nonporous. Figure 1c depicts the Fast Fourier Transformation (FFT) of Figure 1b, which exhibits bright spots along a ring, whose radius will be later compared with XRD results.

**Figure 1:**
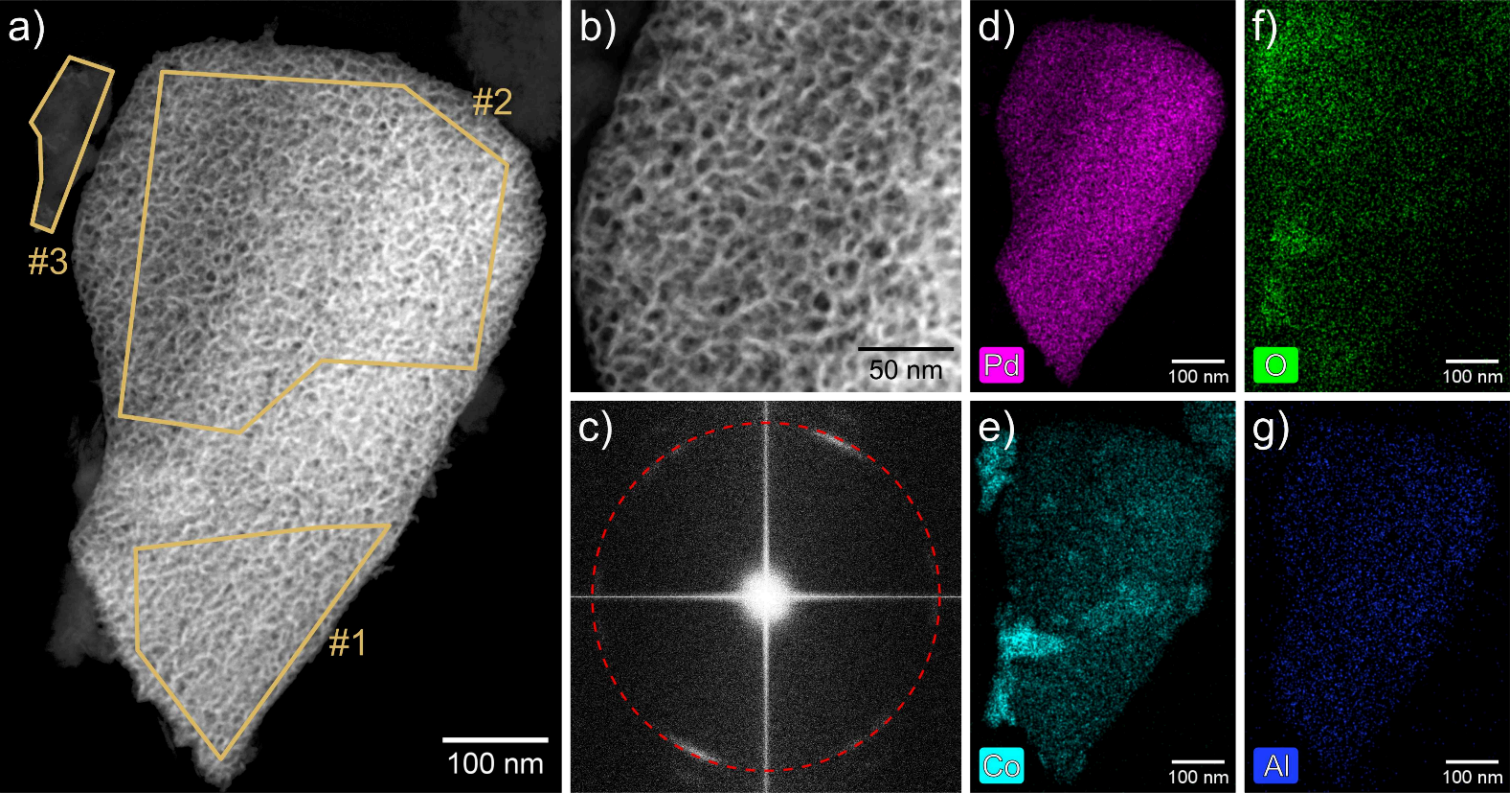
STEM and EDS images of a np Pd_75_Co_25_ sample, produced by dealloying. (a, b) HAADF images with the areas #1 to #3 used for quantitative atomic fraction analysis, (c) FFT of image (b) with red circle marking position of Pd (111) peak in XRD measurement (Figure 2), elemental maps of Pd (pink, d), Co (cyan, e), O (green, f), and Al (blue, g). Note that the contrast in image (a) was enhanced to facilitate the visibility of the nonporous region #3. The original image is shown in Figure S3 in the Supporting Information.

Furthermore, EDS images of the same sample are shown in Figure 1d-g. The elemental map of Pd (Figure 1d) demonstrates a homogeneous distribution in the entirety of the nanoporous areas. Also the distribution of Co (Figure 1e) is found to be uniform in these regions. However, there are some small regions in the sample, like the nonporous area #3 and above the top left corner of area #1, that exhibit a more concentrated Co aggregation. The latter region is most likely influenced by a thin nonporous feature, similar to the one marked as #3, that is located above or below the np sample, leading to the increased Co concentration in this region. Additionally, oxygen is distributed over the whole sample (Figure 1f), exhibiting a clear correlation with the presence of Co. The highest concentrations of oxygen are observed in the regions that are particularly rich in Co. The presence of a small amount of residual Al, distributed throughout the whole particle, was also detected (Figure 1g). Finding such small residual amounts of the sacrificial metal element that are located within the bulk of the porous structure is common for np samples produced by dealloying.^53^

A quantitative analysis of the elemental fraction was conducted for the three regions marked in Figure 1a. The nanoporous areas #1 and #2 both exhibit a Pd to Co ratio of approximately 76 to 24 (see Table S1 in the Supporting Information), which is therefore very close to the nominal ratio in the precursor alloy. The residual amount of the sacrificial metal Al was found to be about 4 atom %. The nonporous region #3 contains mainly Co and O, whereas the atomic fraction of Pd with less than 2 atom % can be neglected. The Al content is even lower than the residual Al content in the nanoporous phases. These low Al and Pd fractions suggest that the nonporous regions were already Co-rich prior to the dealloying procedure, which also explains why no nanoporous structure could evolve by Al dissolution in these regions.

According to the STEM and EDS analysis, the Pd_75_Co_25_ samples prepared by dealloying consist mainly of a np Pd–Co alloy phase with a Co to Pd ratio close to the nominal ratio, and a second nonporous Co-rich phase. To obtain detailed information about the phase composition on the macroscopic scale, XRD was applied, with the resulting data illustrated in Figure 2. The most pronounced peaks, indicated by blue diamonds, are identified as the (111), (200), and (220) peaks of an fcc crystal structure, i.e., Pd. The same lattice pattern is also observed for Pd–Co alloys, where the addition of Co to the Pd structure only leads to a small decrease in the lattice constant.^49,51^ This aligns with the observation that especially the (220), (311), and the (222) peaks demonstrate a slight shift toward higher angles when compared to the Pd reference. It should be noted that the observed diffraction peaks exhibit a relatively large width, which can arise either from the np structure, minor regional variations in the Co/Pd ratio, or lattice deformations.

**Figure 2:**
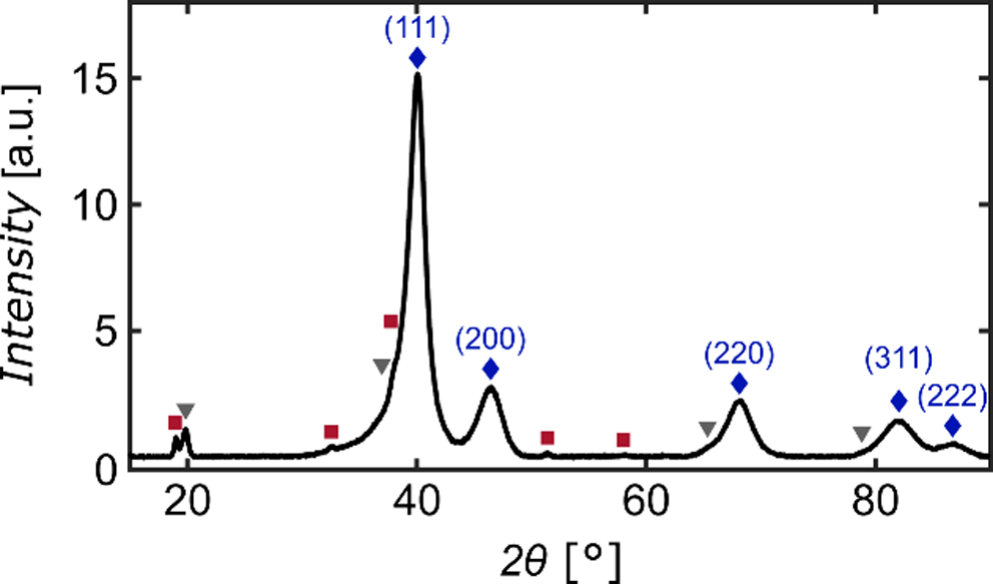
XRD measurement of powdered np Pd_75_Co_25_ sample. Peaks marked with blue diamonds are identified as fcc Pd (PDF 87–0643), red square peaks correspond to hexagonal Co­(OH)_2_ (PDF 30–0443), and peaks with gray triangles are assigned to rhombohedral CoOOH (PDF 07–0169).

The (111) peak exhibits by far the most intense signal of all the observed peaks, and the position of this peak is compared with the FFT analysis of the STEM image of the np structure (Figure 1c) by adding a circle with a radius, matching this peak position. The red circle intersects the visible bright spots, thereby confirming the existence of this crystalline plane, corresponding to the Pd–Co alloy, in the np structure as well.

Besides the dominant Pd–Co alloy phase, the XRD measurement reveals the presence of hexagonal Co­(OH)_2_ (red squares) and CoOOH (gray triangles) phases. This outcome agrees with the observed nonporous Co- and O-rich regions depicted in Figure 1. These peaks are significantly lower than the fcc Pd peaks, indicating that these phases only occur in small amounts, which is consistent with the STEM and EDS investigation.

Additional confirmation of the mainly porous structure was further provided by SEM images (exemplary image in Figure S4 in the Supporting Information), which also revealed only a minor fraction of thin, often hexagonal shaped particles additional to the porous structure. This shape is frequently reported in literature for Co­(OH)_2_,^54,55^ which was also observed in our samples through XRD analysis.

All findings of the structural and elemental analysis confirm the successful formation of a np Pd–Co alloy structure as the predominant phase in our samples with a Co/Pd ratio very close to the nominal ratio. Consequently, the samples will be designated as np Pd_75_Co_25_.

### Electrochemical Characterization

Cyclic voltammetry has been utilized to examine the electrochemical behavior of np Pd_75_Co_25_ in an ex situ setup prior to conducting in situ experiments in the magnetometer. Figure 3 shows the cyclic voltammogram (CV) of np Pd_75_Co_25_ in comparison with CVs of np Pd (Figure 3a) and np Co (Figure 3b) in 1 M KOH, the latter two being produced by dealloying the precursor alloys Al_80_Pd_20_ and Al_80_Co_20_. All three CVs exhibit high gravimetric current densities, which reveals the high surface area of our samples, i.e., their np structure, and additionally indicates a thorough wetting of the samples by the liquid electrolyte. The CV of np Pd_75_Co_25_ exhibits several anodic and cathodic current peaks. The peak positions and overall shape agree well with the CV of np Pd. This allows for the identification of key features by comparing them to the CV of Pd, which has already been studied in numerous works.^56–59^ At positive potentials, a shoulder (I_*a*_) appears, indicating that Pd oxides are formed at the surface of the np Pd_75_Co_25_ sample, which are subsequently reduced at the cathodic current peak I_*c*_. Close to the upper potential limit, the oxygen evolution reaction causes a rapid current increase. The cathodic peak II_*c*_ at – 0.63 V in the CV of Pd_75_Co_25_ can be attributed to the adsorption of hydrogen onto the sample surface, and the anodic peak II_*a*_ at – 0.33 V to the removal of the hydrogen adsorbed at the surface. The clear occurrence of these hydrogen adsorption and desorption peaks is consistent with the np structure of our samples, as this is typical for Pd samples with a high surface to volume ratio.^58,60^ In comparison to the corresponding peaks in the CV of Pd, these peaks are shifted to more positive potentials for our Pd_75_Co_25_ alloy, which could arise from a lower energy necessary to adsorb H on Pd–Co alloys compared to Pd, as indicated by recent DFT calculations.^61^

**Figure 3:**
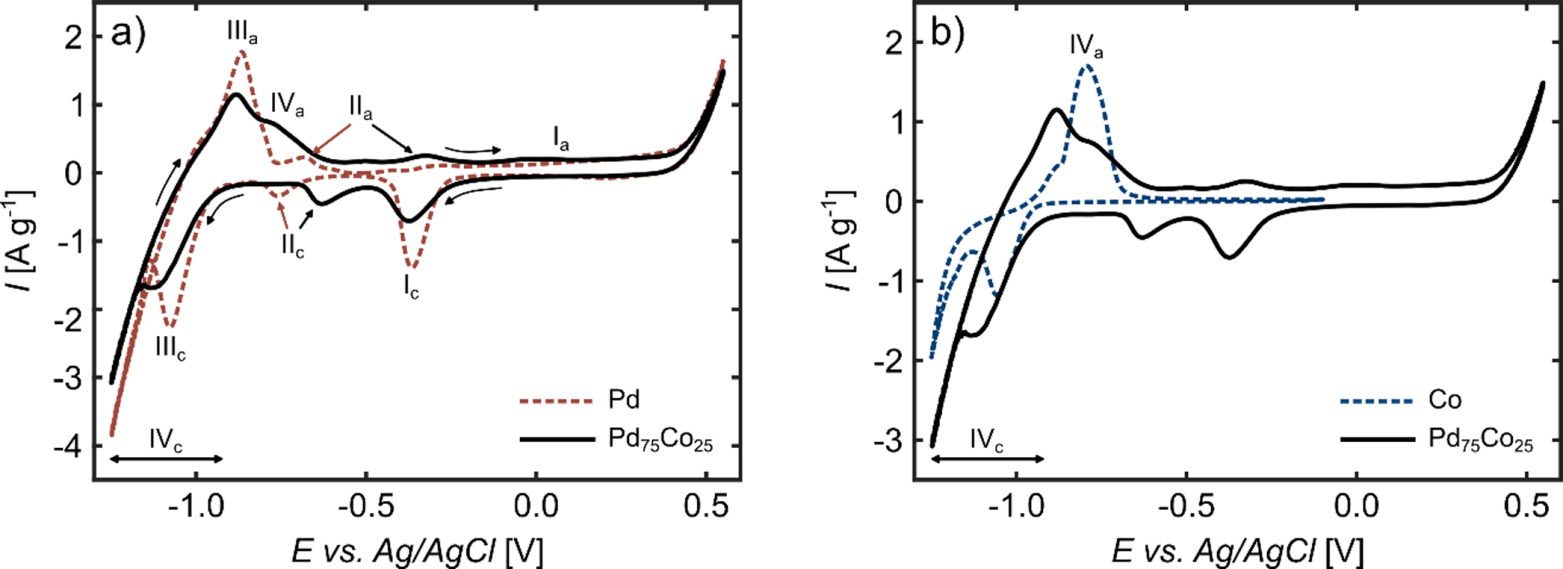
Cyclic voltammograms of (a) np Pd_75_Co_25_ in comparison with np Pd and (b) np Pd_75_Co_25_ in comparison with np Co, measured between the potential limits of −1.25 and 0.55 V at a scan rate of 0.5 m V s^–1^ in 1 M KOH. The Roman numerals indicate the main features of the CVs, which are described in detail in the text.

The cathodic peak III_*c*_ can be ascribed to the hydrogen absorption. It is positioned at roughly the same potential as for np Pd, with the corresponding desorption reaction occurring at the anodic peak III_*a*_. The strong cathodic current increase for more negative potentials is caused by the hydrogen evolution reaction (HER). The appearance of a peak for hydrogen absorption, instead of a continuous current increase, which is superimposed by the current of the HER, is a further characteristic known for Pd electrodes with a high surface to volume ratio.^56,58,60^ The peak indicates that the sample becomes already saturated with hydrogen during the CV measurement, when the peak is exceeded.^58^ The lower amplitude of the peaks III_*c*_ and III_*a*_ compared to pure Pd fits with the observation that less H can be incorporated into the alloy.^62^

In addition to the features that overlap with those of the Pd CV, there are some that only appear for the Pd_75_Co_25_ alloy. The hydrogen desorption peak III_*a*_ is superimposed by a second peak IV_*a*_ and the hydrogen absorption peak III_*c*_ is less sharply defined, indicating an additional reversible electrochemical reaction in this potential range. Because of the significant Co content in our np Pd_75_Co_25_ samples, an appearance of current features in the CV due to electrochemical reactions of Co is expected. We thus compared in Figure 3b the same CV of np Pd_75_Co_25_ as in Figure 3a with a CV of np Co. It immediately becomes clear that the CV of the Co sample exhibits current peaks at exactly these aforementioned potentials of np Pd_75_Co_25_. Based on the investigation of Behl et al.^63^ on Co in potassium hydroxide electrolytes, the anodic (IV_*a*_) and cathodic current (IV_*c*_) peaks in the CV of Co can be ascribed to the oxidation of Co to Co­(OH)_2_ and CoO, and the reduction back to metallic Co. The negative current values across the entire potential range, specified by the arrow labeled as IV_*c*_ in both scan directions, indicates a persistent reduction of Co occurring even in the anodic scan. Consequently, the comparison with the Co CV reveals that the anodic peak IV_*a*_ of Pd_75_Co_25_ can be attributed to an oxidation of Co, whereas the reduction of Co (hydr-)­oxides (IV_*c*_) takes place concurrently with the hydrogen absorption (III_*c*_) and also continues in the potential region of the HER and at the beginning of the anodic backscan.

In order to gain a more in-depth understanding about the potential region where the H absorption/desorption and the reduction/formation of Co (hydr-)­oxides takes place, additional cyclic voltammetry measurements were conducted. The lower potential limit was varied for both the np Pd_75_Co_25_ sample (Figure 4a) and the np Pd sample (Figure 4b), while the upper potential limit was kept constant at – 0.5 V. As demonstrated in Figure 4b, there is a clear correlation between the hydrogen desorption peak III_*a*_ and the cathodic peak III_*c*_ (hydrogen absorption) for the Pd sample. It becomes evident that the desorption peak does not increase in size, when the lower voltage limit is varied from −1.15 V to the more negative value of −1.25 V. This indicates that indeed a saturation of the hydrogen concentration in the sample takes place over the course of the hydrogen absorption peak, meaning that an application of more negative potentials does not result in further intercalation of hydrogen. The CVs of np Pd_75_Co_25_ in Figure 4a exhibit the same behavior, when looking at the anodic peak III_*a*_. The peak height and area beneath the peak remain unaltered when the lower potential limit is varied from −1.15 V to −1.25 V. However, the Co oxidation peak (IV_*a*_) becomes more prominent, when a more negative lower potential limit is selected. Based on this electrochemical characterization of np Pd_75_Co_25_, it can thus be concluded that, at the investigated scan rate, a lower voltage limit of −1.15 V is sufficient to complete the hydrogen absorption. Conversely, the Co reduction, which also occurs throughout the hydrogen absorption reaction, proceeds at more negative potentials, where also the HER takes place.

**Figure 4:**
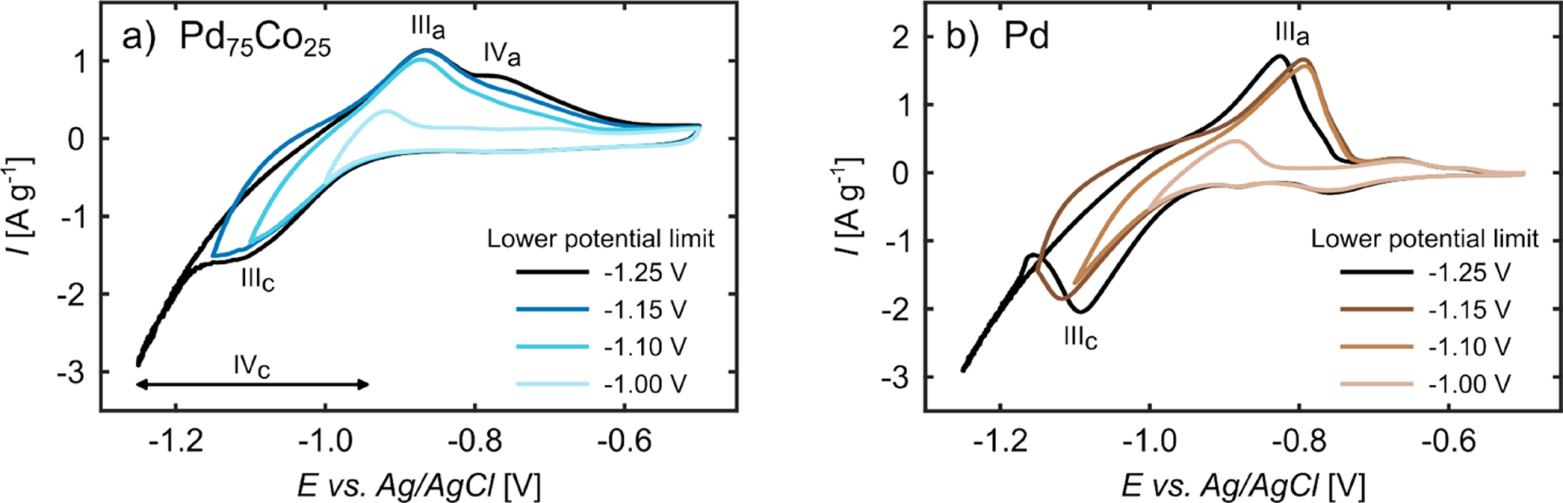
Cyclic voltammograms with variable lower potential limits of (a) np Pd_75_Co_25_ and (b) np Pd in 1 M KOH, measured with a scan rate of 0.5 m V s^–1^ and an upper potential limit of −0.5 V. The Roman numerals indicate the main features of the CVs, which are described in detail in the text.

### Characterization of the Initial Magnetic State

The initial magnetic state of np Pd_75_Co_25_ was determined by measuring the magnetic moment as a function of the applied magnetic field. In addition, ZFC/FC curves were recorded. Both results are illustrated in Figure 5. The magnetization curve, measured at 300 K (Figure 5a), exhibits no hysteresis splitting and indicates a weak superparamagnetic behavior (saturation magnetization of just about 0.5 emu g^–1^) with an additional paramagnetic slope. The presence of a superparamagnetic state at room temperature is further supported by the ZFC/FC curves, where the ZFC branch shows a characteristic blocking peak at a temperature of about 30 K, representing the transition from a superparamagnetic to a ferromagnetic state (Figure 5b). This is also reflected in the splitting of the ZFC and FC curves at lower temperatures. Since the np Pd_75_Co_25_ alloy of the selected composition is expected to exhibit ferromagnetic properties at room temperature, the occurrence of the very weak magnetic signal, consisting of a superparamagnetic and paramagnetic contribution, is an evidence that this sample is oxidized in the initial state, as already seen in the elemental analysis. Co oxides and hydroxides, such as CoO, Co_3_O_4_, CoOOH, and Co­(OH)_2_ are paramagnetic at 300 K.^64–67^ The weak superparamagnetic signal might be attributed to very small regions in the sample that are not fully oxidized.

**Figure 5:**
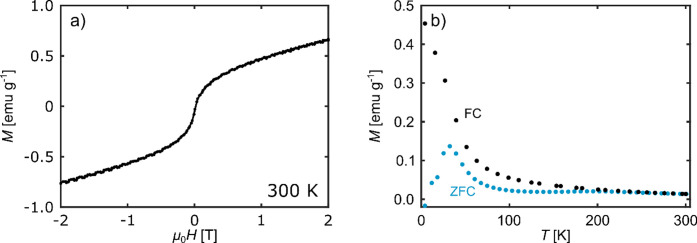
Characterization of the initial magnetic state of np Pd_75_Co_25_. (a) Magnetization curve recorded at 300 K with a VSM. (b) ZFC/FC measurement taken at a magnetic field of 0.005 T between 4.2 and 300 K.

### Magneto-ionic Effects

#### Main Effect Based on Co (Hydr-)­oxide Reduction/Formation

SQUID magnetometry measurements were combined with in situ cyclic voltammetry to monitor changes in the magnetization of np Pd_75_Co_25_ simultaneously with the occurring electrochemical processes. The result in Figure 6 shows a substantial increase in magnetization during potential cycling, which was performed between the potentials of 0 V and −1.225 V, i.e., in the potential region where the H absorption/desorption and the reduction/formation of Co (hydr-)­oxides takes place (see Figure 3). As shown in the Supporting Information (see black curves in Figure S5), no significant additional magnetization variations occur when the potential range is further extended to the regime of Pd oxide formation and reduction. The cycle in Figure 6a was initiated at an open circuit potential (OCP) of approximately −0.25 V, with an initial magnetization of just 0.4 emu g^–1^, a value that is consistent with the ex situ characterization of the initial magnetic state. Upon cycling to more negative potentials, the magnetization starts to increase slowly at approximately −0.8 V, i.e., in the potential region where the H absorption as well as the reduction of Co (hydr-)­oxides set in. The increase in magnetization becomes more pronounced as the potential reaches more negative values, and persists when the H absorption peak is exceeded. Subsequent to the reversal of the scan direction, the magnetization continues to rise, however, with a gradient that declines slightly but continuously (see Figure 6b). The magnetization still increases when reaching the hydrogen desorption peak in the anodic scan (see Figure 6a) and attains a maximum of 11.9 emu g^–1^ at a potential of about −0.7 V. It should be noted that, as shown in Figure S5 in the Supporting Information, the maximum value of the magnetization obtained during such a CV measurement depends on the lower potential limit of the cycle. As the cycle progresses toward more positive potentials, a rapid decline in magnetization becomes evident, until eventually the initial magnetization value is reached again. Comparing the decrease in magnetization and the shape of the cyclic voltammogram, it becomes obvious that the decrease commences at the onset of the Co oxidation peak and becomes the fastest at the peak maximum, i.e., when the highest oxidation rate is attained (see also CV in Figure 3b). Overall, the correlations between the magnetization variations and the electrochemical processes identified by the in situ CV measurements clearly show that the substantial magnetism changes observed are mainly caused by the formation/reduction of Co (hydr-)­oxides rather than the absorption/desorption of hydrogen.

**Figure 6:**
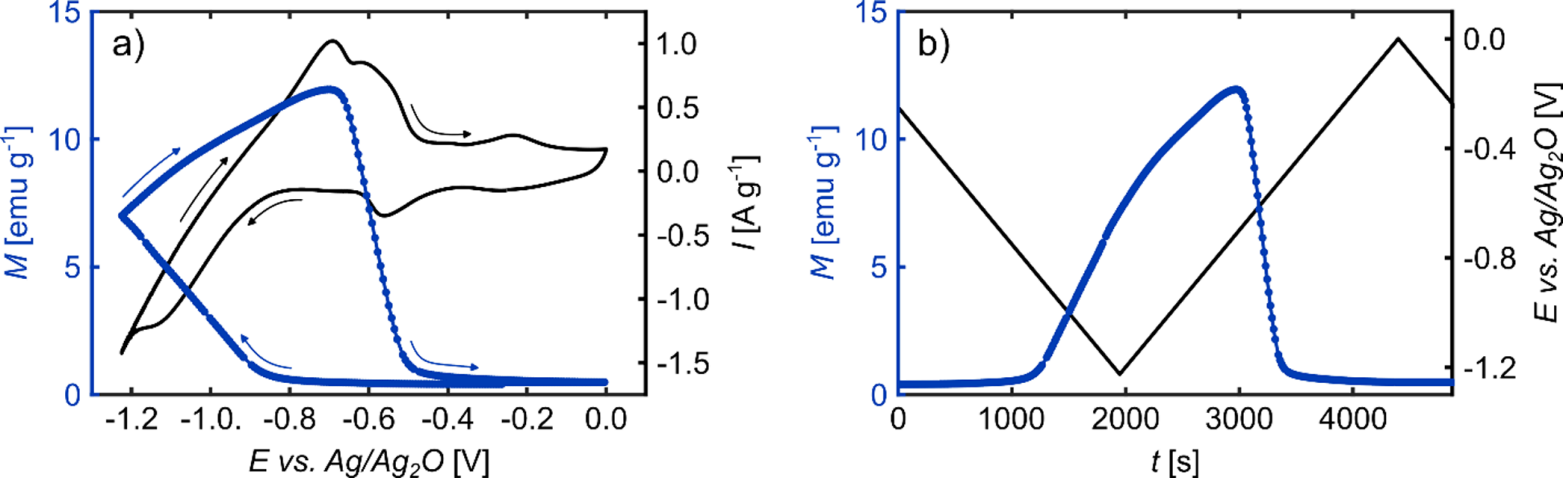
Magnetization change during cyclic voltammetry of np Pd_75_Co_25_ in 1 M KOH at a scan rate of 0.5 m V s^–1^. (a) Electric current *I* and magnetization *M* as a function of the applied potential *E* during cycling between 0 V and −1.225 V. (b) *M* and *E* of the measurement in (a) as a function of time *t*.

A deeper insight into the magnetic states of our np Pd_75_Co_25_ samples during potential cycling was provided by measuring magnetization curves at specific potentials, as it can be seen in Figure 7. To achieve this, a new cyclic voltammetry experiment was conducted, where the potential sweeping was stopped in the anodic scan at −0.7 V near the maximum of the magnetization, and at the upper potential limit of 0 V after the magnetization decreased again to the initial value. At the beginning of this measurement, a magnetization curve was also recorded at the open circuit potential, prior to any potential application. As shown by the black curve, the np Pd_75_Co_25_ exhibits a weak superparamagnetic behavior with a small overlaying paramagnetic contribution at this initial state. This outcome aligns well with the ex situ determination of the initial magnetic state depicted in Figure 5. In contrast to the initial state, the red magnetization curve recorded at −0.7 V shows a significantly increased saturation magnetization and a clear ferromagnetic hysteresis splitting. The occurrence of ferromagnetism is expected for Pd–Co alloys of this composition, and is therefore another indication that the np Pd_75_Co_25_ sample is, at least partly, in a reduced, metallic state at this potential. The magnetization curve recorded afterward at the upper potential limit of 0 V (blue) shows again a significantly reduced magnetic response, corresponding to a magnetic OFF state. In fact, the saturation magnetization at this potential is even smaller than in the initial condition recorded at OCP. This is most probably due to a stronger oxidation of the sample resulting from the application of more positive potentials. All in all, the magnetization curves reveal that applying an external voltage to the np Pd_75_Co_25_ alloy does not only lead to reversible variations of the magnetic moment, but also to an ON/OFF switching of magnetism between a ferromagnetic ON state and a weakly magnetic OFF state.

**Figure 7:**
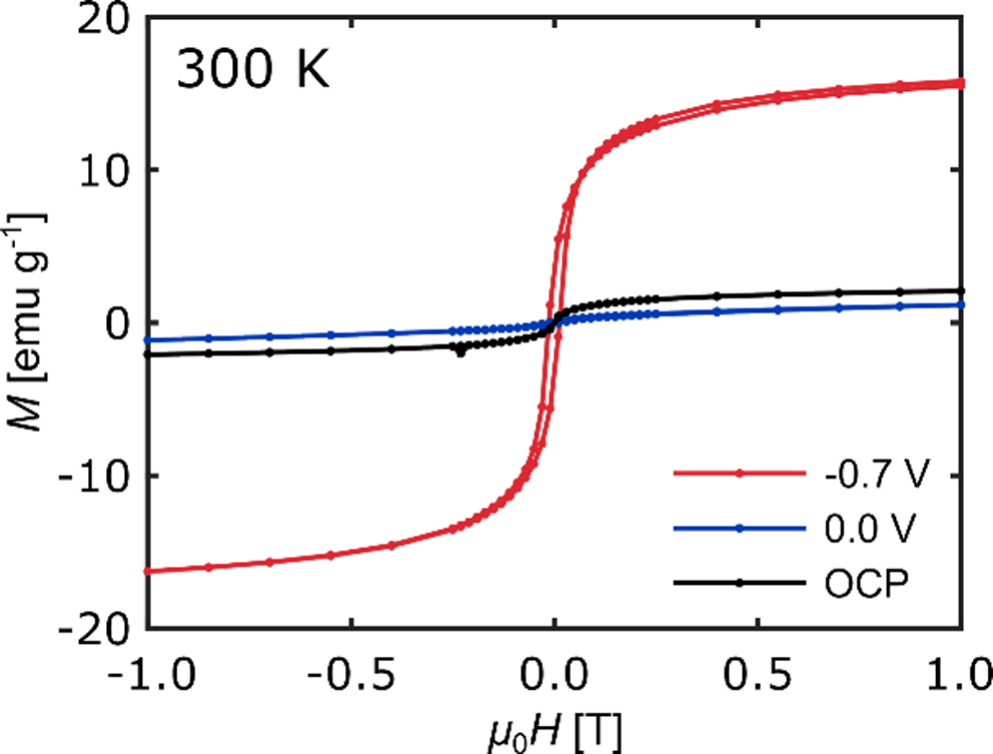
Magnetization curves of np Pd_75_Co_25_ at 300 K measured in situ at the initial open circuit potential (OCP, black), at – 0.7 V (red) in the anodic scan direction, and at 0 V (blue) vs Ag/Ag_2_O. Note that the curve at – 0.7 V was corrected for a magnetization drift occurring during the measurement.

#### Additional Influence of H Absorption/Desorption

Based on the previous measurement results, it was concluded that the reduction/formation of Co (hydr-)­oxides, rather than the absorption/desorption of hydrogen is the primary cause of the significant voltage-induced variations in magnetism encountered for the np Pd_75_Co_25_ alloy. Here, further evidence for this conclusion will be presented, but it will also be revealed that the magnetization of np Pd_75_Co_25_ is to some extent affected by the absorption/desorption of H. To better separate these two reactions, which partly overlap in the CV, additional in situ measurements were conducted in the SQUID magnetometer, where a potential holding step was incorporated.

As illustrated in Figure 8a, the initial phase (i) of the experiment comprises a cyclic voltammogram that is halted in the anodic scan at a potential of −0.95 V. In the second phase (ii), this potential is kept constant for 45 min. Subsequently, the cyclic voltammetry measurement is continued from this potential in the third phase (iii) of the experiment. In Figure 8b, the CV of this third phase is shown in beige together with the corresponding magnetization variation. Moreover, for comparison, the data of a regular full cycle without any prior potential holding is included in this graph as black curves.

**Figure 8:**
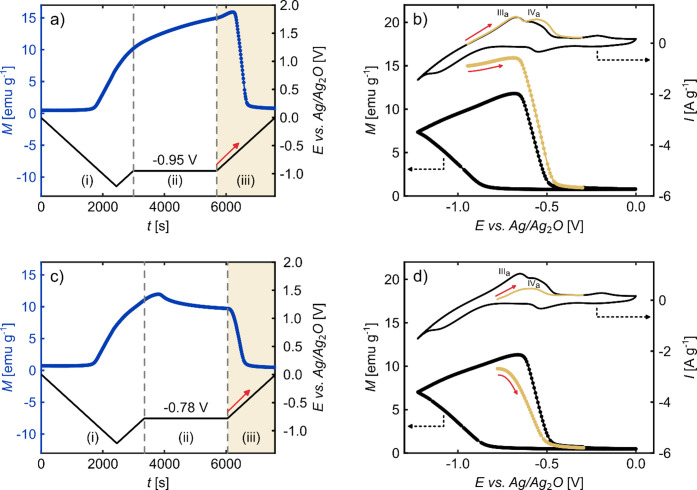
Investigation of magneto-ionic effects of np Pd_75_Co_25_ by combining cyclic voltammetry with a scan rate of 0.5 m V s^–1^, potential holding, and SQUID magnetometry at 300 K. (a, c) Variation of the applied potential *E* and measurement of the corresponding magnetization *M* during potential cycling (i, iii) and potential holding (ii), plotted as a function of time *t*. (b, d) CV and corresponding *M* variation of phase (iii) in beige are compared to a regular cycle in black without prior potential holding. See text for further details.

As depicted in Figure 8a, the behavior of the magnetization in the first phase (i) of the experiment is identical to the one observed in the previous measurement (see Figure 6b). The magnetization shows a continuous increase, starting at approximately −0.8 V in the cathodic scan (1600 s on the time axis). When holding the potential at −0.95 V in the second phase (ii), the increase of *M* continues, but with a diminishing gradient over time. This implies that the reaction responsible for the magnetization increase is still occurring during the potential holding step. This reaction can be identified by comparing the CV of the third phase (iii, beige) with the regular CV (black) in Figure 8b. Evidently, the hydrogen desorption peak (III_*a*_) remains unchanged, while the Co oxidation peak (IV_*a*_) is significantly enhanced after including the potential holding step (ii). This indicates that no further hydrogen absorption occurred during the potential holding step; however, the reduction to metallic Co continued. Consequently, these findings provide additional proof that the increase in the magnetization of np Pd_75_Co_25_ during potential cycling is primarily the result of the reduction of (hydr-)­oxides, rather than the absorption of hydrogen.

A closer look at phase (iii) in Figure 8a reveals that the magnetization increase at the beginning of the resumed CV measurement is more pronounced than during the potential holding step before. Since the Co reduction rate decreases when going to more positive potentials, a faster Co reduction cannot be the cause of this increased slope. As can be seen from Figure 8b (beige data), also the hydrogen desorption takes place in this potential regime. Akamaru et al.^42^ have reported that the absorption of hydrogen into Pd–Co alloys results in a decrease in magnetization due to changes in the electronic structure, which is subsequently followed by an increase during the desorption. Thus, it can be assumed that the desorption of hydrogen from our np Pd_75_Co_25_ alloy is the reason for this additional magnetization increase. It should be mentioned that structural effects induced by H incorporation, such as additional defects and induced stresses, are not expected to have a significant impact on the magnetization, because they primarily affect the coercivity of Pd–Co alloys rather than the saturation magnetization.^68^

Adding the potential holding step to the CV results in a maximum magnetization of 15.9 emu g^–1^ (see Figure 8a), which exceeds the value obtained in the previous CV measurement (see Figure 6). This value corresponds to approximately one-third of the expected saturation magnetization of a Pd_75_Co_25_ alloy, estimated from the data of Bozorth et al.^51^ to be around 48.5 emu g^–1^. One reason for the lower value obtained for our np Pd_75_Co_25_ alloy is that, even after the end of the additional potential holding step, the magnetization continues to increase, which indicates that our sample is not in a fully reduced state at this point. Therefore, even higher magnetization values could be obtained by adapting the measurement procedure further, such as increasing the holding time or applying more negative potentials. However, the maximum achievable magnetization value is expected to be somewhat lower than 48.5 emu g^–1^, since a complete reduction cannot be anticipated throughout the entire sample. This is especially the case for the nonporous Co-rich regions. Additionally, the presence of small amounts of residual Al in our np sample is highly likely another cause for a reduced magnetization value, as Al is known to have a decreasing effect on the magnetization when added to pure Co and Co alloys.^69–71^

To further elucidate the influence of the hydrogen absorption/desorption on the magnetization, a second experiment was performed (Figure 8c,d), where the cyclic voltammogram of the initial phase (i) was stopped in the anodic scan at a more positive potential of −0.78 V. This potential lies already in the onset of the hydrogen desorption peak (III_*a*_), i.e., hydrogen desorption should take place during the potential holding phase. Moreover, it is assumed that no further reduction of Co (hydr-)­oxides occurs at this potential, since it is more positive than the onset potential of this process. As can be seen in Figure 8c, a different behavior of the magnetization during potential holding (ii) was observed, compared to the previous experiment. In the initial part of phase (ii), the magnetization increases; however, it then decreases again until the end of the constant potential step. This finding indicates the occurrence of two processes with opposing effects on the magnetization at this constant potential. Based on the shape of the cyclic voltammogram of phase (iii) in Figure 8d (beige), where the hydrogen desorption peak (III_*a*_) is not observed, it can be concluded that all of the previously absorbed hydrogen left the sample during the potential holding step (ii). The complete removal of the hydrogen from the sample is consistent with the observed increase in magnetization at the beginning of phase (ii). The subsequent decline in magnetization during the potential holding phase suggests that a slight Co oxidation already takes place during this step. This is confirmed by the fact that the following CV (beige in Figure 8d) immediately starts with the Co oxidation peak (IV_*a*_) and the corresponding magnetization decrease, and that both are reduced in their magnitude compared to the regular cycle (black data).

Overall, it can be concluded from this measurement series that, although the magnetization of our np Pd_75_Co_25_ samples is mainly influenced by the reduction/formation of Co (hydr-)­oxides, there is also a distinct impact of the hydrogen absorption/desorption process on the magnetization of the samples. In agreement with literature,^42^ the absorption and desorption of hydrogen yield a decrease and increase in the magnetization, respectively. However, the influence of hydrogen is weaker, and thus only becomes discernible when the reduction/formation of the Co (hydr-)­oxides is not strongly pronounced, as is observed in certain phases during the experiments with potential holding steps (see Figure 8a,c). For the conventional potential cycling experiment of Figure 6, the impact of the hydrogen absorption/desorption is predominantly masked by the substantial magnetization variations arising from the reduction/formation of Co (hydr-)­oxides. The following more detailed description can be given for the observed magnetization variations. As schematically illustrated in Figure 9, the significant increase in magnetization during the cathodic scan is due to the reduction of Co (hydr-)­oxides (see red part). Due to this strong magnetization change, there is no visible impact of the simultaneous absorption of hydrogen, which should lead to a magnetization decrease. In the anodic scan, the magnetization increase that occurs after the scan direction is reversed can be explained by the ongoing (hydr-)­oxide reduction. This process becomes less pronounced when going to more positive potentials, however, here the contribution of the hydrogen desorption to the magnetization increase sets in and becomes after some time the dominant effect (see blue part). The rate of increase slows down when the onset of the Co oxidation peak is reached, and the magnetization decreases rapidly as soon as the Co oxidation becomes the dominant effect (see green part). The magnetization reaches its approximate initial value by the time the Co oxidation peak is completed.

**Figure 9:**
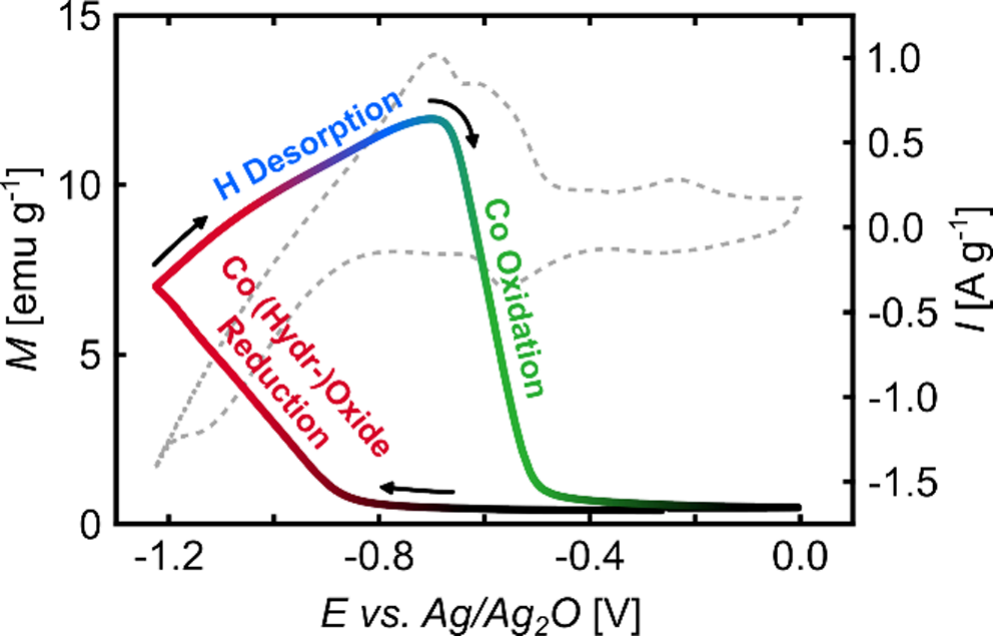
Magnetization *M* during the cyclic voltammetry experiment of Figure 6. The different colors schematically indicate which process is mainly responsible for the magnetization variation.

Finally, it should be emphasized that, although the influence of the hydrogen absorption/desorption on the magnetization of our np Pd_75_Co_25_ sample is mainly superimposed by the stronger variations due to the Co (hydr-)­oxide reduction/formation, it seems to be promising to further enhance this hydrogen based magneto-ionic effect by adapting the alloy composition. Because increasing the Pd fraction of the alloy should not only allow for more hydrogen to be incorporated,^62^ but also larger relative magnetism variations were observed for Pd–Co alloys with higher Pd fractions by hydrogen absorption from the gas phase.^42^

#### Dynamics of Magneto-ionic Effects

The dynamics of the voltage-induced magnetization changes were analyzed through potential jumps, which can be seen in Figure 10a. First, a negative potential of either – 0.9 V, – 1.0 V, or – 1.1 V was applied for 5 min. According to the CVs (Figure 3), these potentials lie in the regime of Co (hydr-)­oxide reduction and hydrogen absorption, with increasing reaction rates for more negative potentials. Subsequently, a potential of 0 V was applied for the same period of time, at which Co oxidation and H desorption are known to take place.

**Figure 10:**
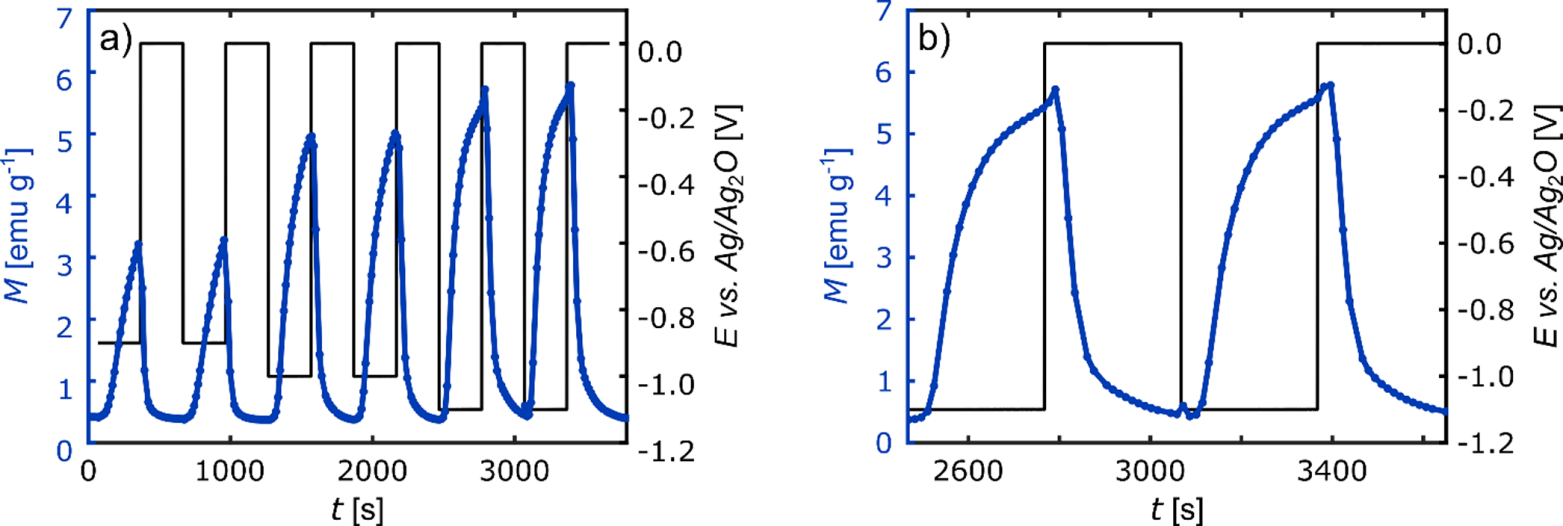
(a) Potential jumps with np Pd_75_Co_25_ between *E* = −1.1 V, −1.0 V, −0.9 V, and 0 V in 1 M KOH with the associated magnetization variation *M*. After every jump, the potential was applied for a time *t* of 300 s. (b) Detailed representation of potential jumps between −1.1 and 0 V of measurement in (a).

As demonstrated in Figure 10, the magnetization exhibits a continuous increase in response to the application of the negative potentials, with a more pronounced and accelerated increase for more negative potentials. Consequently, for a more negative applied potential, it is possible to either achieve a larger magnetization during an equivalent application time, or to achieve the same magnetization in a shorter time period. It is notable that, especially at −1.1 V, the slope of the magnetization declines after some time, which indicates a slowdown in the rate of Co reduction. When applying 0 V, the magnetization decreases in all cases again back to its initial value within the 5 min step. All three potential jumps were repeated for a second time, yielding the same magnetization change as for the first jumps. The high reversibility suggests that this magneto-ionic system could enable a stable switching over many cycles. However, further long-term cycling experiments will be necessary to confirm this assumption.

An interesting observation can be made when examining Figure 10b more closely, namely that the first two magnetization data points following the potential jump from −1.1 to 0 V demonstrate higher values in comparison to the last data points recorded at −1.1 V. This behavior is present in both measurements and can again be explained by the overlapping of the hydrogen desorption, leading to a magnetization increase, and the Co oxidation, leading to the decline of the magnetization. The immediate small increase after jumping to 0 V implies that the hydrogen desorption is the much faster process, occurring on a time scale of seconds, whereas it takes a few minutes until the oxidation of Co is concluded. The time scale of hydrogen desorption in the range of seconds implies that the hydrogen diffusion in the Pd_75_Co_25_ alloy is not the rate limiting step, since this process can be estimated to take place within microseconds using the hydrogen diffusion coefficient for Pd_75_Co_25_ of 3 × 10^–12^ m^2^ s^–1^ ^72^ and assuming a diffusion length of 5 nm. However, the time scale aligns with reported electrochemical hydrogen desorption times for np Pd, where the proton transport within the electrolyte was identified as the rate-controlling step.^73^ Altogether, this measurement reveals that, in our np Pd_75_Co_25_ samples, the hydrogen magneto-ionic effect is significantly faster than the (hydr-)­oxide magneto-ionic effect. It should be noted that the rise of the magnetization at the beginning of the 0 V step was not observed for the other potential jumps, which could be due to a lower hydrogen concentration resulting from the application of less negative potentials (−0.9 V, – 1 V), diminishing the hydrogen effect or making it so short that it can not be observed anymore with the SQUID magnetometer, which is capable of recording only a single data point every 14 s.

In summary, the present experiment reveals that with our np Pd_75_Co_25_ samples it is possible to obtain substantial reversible variations of the magnetization in the time frame of a few minutes, which can be attributed to their high surface-to-volume ratio. As the samples under investigation are macroscopic in their dimension, these variations also correspond to significant changes in the absolute magnetic moment, which are in the order of 0.01 emu. It is noteworthy that the magnetization continued to increase at the end of the 5 min negative potential application, which implies that even larger variations could be achieved by increasing the time. Based on the progression of the magnetization curve during the – 1.1 V step, it can be anticipated that magnetization values higher than 10 emu g^–1^, as obtained during previous measurements, can be reached within about 30 min.

## Conclusion

In this work, a np Pd_75_Co_25_ alloy was successfully prepared through a process of dealloying, leading to pore sizes of around 10 nm. In situ electrochemical measurements in a SQUID magnetometer have demonstrated that the magnetization of the alloy can be switched in a reversible manner between a ferromagnetic ON state, exhibiting a high absolute magnetic moment in the order of 0.01 emu, and an OFF state, in which the magnetization is significantly reduced, in the time frame of just several minutes. A detailed electrochemical investigation revealed that the significant magnetization variation can be ascribed mainly to the formation and reduction of Co oxides and hydroxides, thus to a magneto-ionic effect based on oxygen species. Moreover, an additional magneto-ionic effect occurs in np Pd_75_Co_25_ that can be attributed to the absorption/desorption of hydrogen. This second effect is smaller in size, and since the absorption process takes place simultaneously with the Co (hydr-)­oxide reduction, the effect is only prominent during the hydrogen desorption process, when an additional increase in magnetization takes place in the time frame of just a few seconds.

Overall, it was demonstrated for the first time that magneto-ionic effects based on two different kinds of ions can be obtained at room temperature in an alloy sample. This is particularly attractive, because it allows for adjusting the magneto-ionic response by adapting the alloy composition. For Pd–Co alloys, it can be expected that a reduction of the oxophilic Co content, coupled with an increase in the atomic fraction of the hydrophilic Pd, results in enhanced hydrogen absorption and an amplified hydrogen based magneto-ionic effect, while simultaneously the effect based on the formation and reduction of Co oxides and hydroxides should be reduced. First indications of this behavior were already found during preliminary measurements on samples with different compositions. Considering the different influence of the two magneto-ionic effects on the magnetization and their different dynamics, this adaptability opens up the way to engineer the magneto-ionic response of the alloy system, which could be especially attractive to mimic various neuromorphic functions.

## Supplementary Material



## Notes

Open circuit potential measurement of a Ag/Ag_2_O quasi-reference electrode, schematic illustration of the cell setup for in situ electrochemical measurements in the SQUID magnetometer, original STEM image of Figure 1a without contrast enhancement, table containing the quantitative STEM/EDS analysis of Figure 1a, SEM image of the surface of a np Pd_75_Co_25_ sample, and cyclic voltammetry measurement of a np Pd_75_Co_25_ sample with variable potential limits and the corresponding magnetization variation (PDF)
