# Supplementary material for: Dual-Ion Based Magneto-ionic Effects in Nanoporous Pd75Co25 Alloy
Source: ACS Mater Au. 2026 Mar 20;6(3):583–95. doi: 10.1021/acsmaterialsau.5c00245 (PMC13177409; doi:10.1021/acsmaterialsau.5c00245)
Supplement: Supplementary file 1 [file mg5c00245_si_001.pdf]

Supporting Information

Dual-Ion Based Magneto-ionic Effects in Nanoporous $Pd_{75}Co_{25}$ Alloy

Stefan Eber[1*], Georg Haberfehlner[2], Peter Banzer[3], Roland Würschum[1], Stefan Topolovec[1*]

Author Affiliations

[1]Institute of Materials Physics, Graz University of Technology, NAWI Graz, Petersgasse 16, 8010 Graz, Austria

[2]Institute of Electron Microscopy and Nanoanalysis, Graz University of Technology, NAWI Graz, Steyrergasse 17, 8010 Graz, Austria

[3]Institute of Physics, University of Graz, NAWI Graz, Universitätsplatz 5, 8010 Graz, Austria

[*]E-mail: S.E. (stefan.eber@tugraz.at), S.T. (stefan.topolovec@tugraz.at)

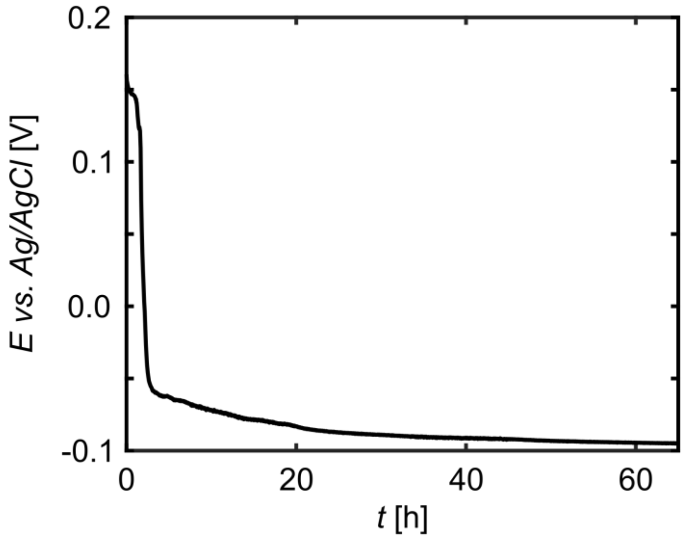

Figure S1: Potential *E* of Ag/Ag_2O quasi-reference electrode in 1 M KOH with respect to a Ag/AgCl reference electrode during measurement of the open circuit potential as a function of time *t*. It should be mentioned that measurements utilizing a Ag/Ag_2O electrode as reference were started no earlier than 12 h after immersing it in the electrolyte, to ensure that any potential drift in the reference potential is minimized.

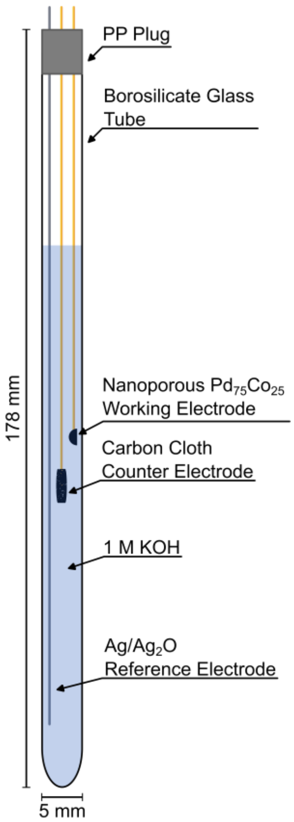

Figure S2: Cell design for in situ electrochemical experiments in SQUID magnetometer, consisting of a borosilicate glass tube with three electrodes and a 1 M KOH aqueous solution.

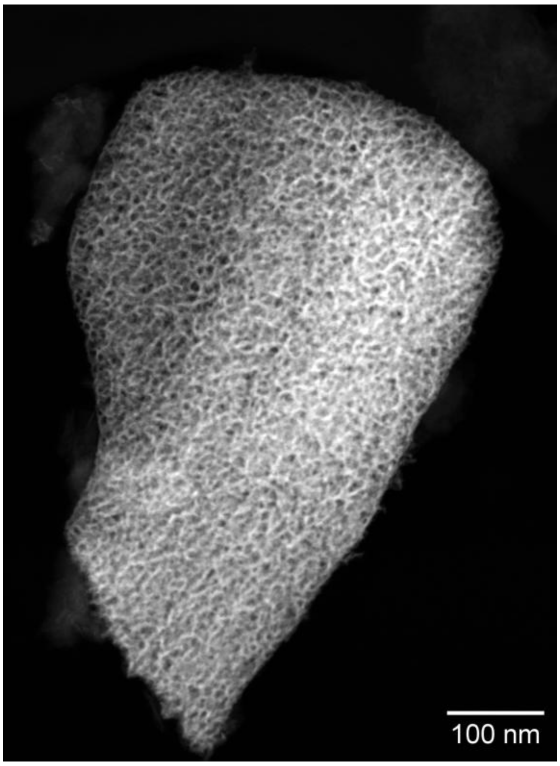

Figure S3: Original STEM image (HAADF) of a np $Pd_{75}Co_{25}$ sample, shown in Figure 1a, without contrast enhancement.

Table S1: Quantitative elemental analysis of Pd, Co, O, and Al in the areas #1, #2, and #3 of the STEM/EDS image in Figure 1a.

	Atomic fraction [%]			
	**Pd**	**Co**	**O**	**Al**
**Area #1**	62.5	19.0	14.5	4.0
**Area #2**	58.7	18.5	18.7	4.1
**Area #3**	1.5	40.6	57.1	0.8

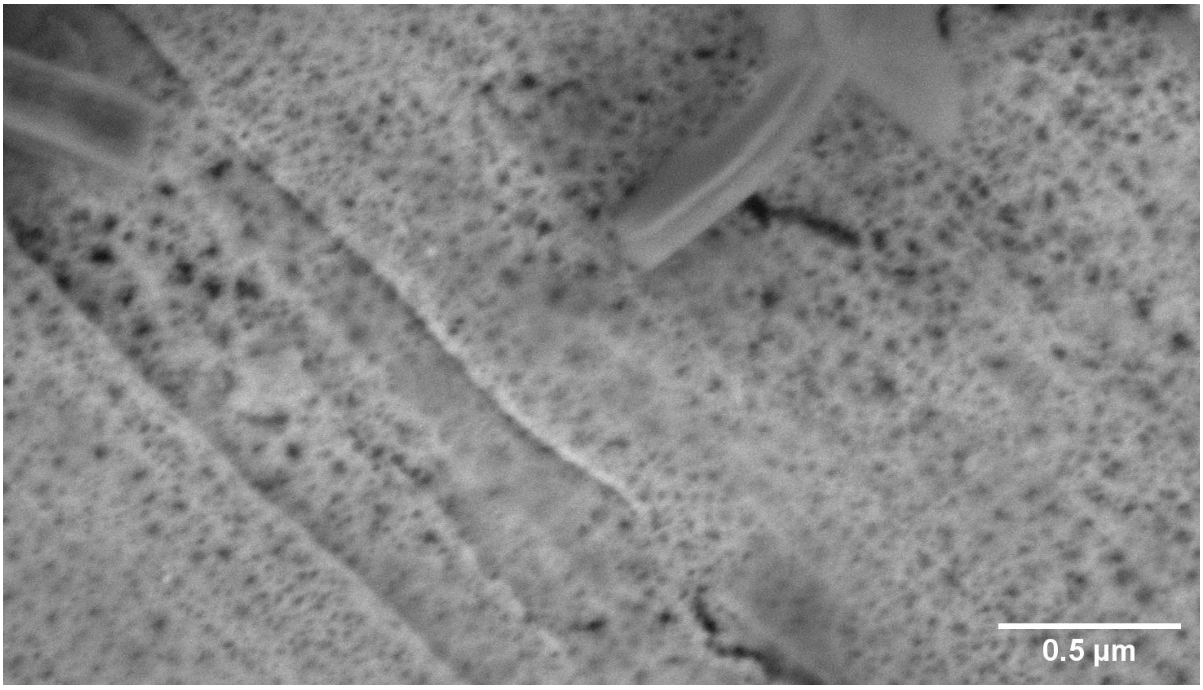

Figure S4: SEM image of np $Pd_{75}Co_{25}$ with a well visible porous structure and thin, plate-like particles on the surface.

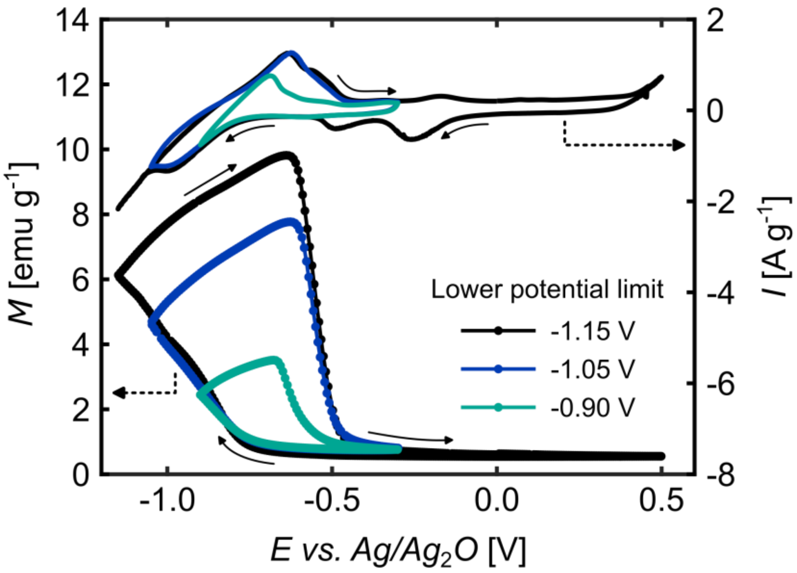

Figure S5: Magnetization *M* of np $Pd_{75}Co_{25}$ during cyclic voltammetry with variable potential limits in 1 M KOH and a scan rate of 0.5 mV/s. The black curves show a cyclic voltammogram and the corresponding magnetization changes in the potential window of -1.15 V and 0.5 V. No significant magnetization changes can be observed in the positive potential region. Comparing all three measurement procedures, it can be concluded that a more negative lower potential limit results in a higher maximum magnetization.
